# Risk for Behavioral Problems Independent of Cognitive Functioning in Children Born at Low Gestational Ages

**DOI:** 10.3389/fped.2020.00311

**Published:** 2020-06-26

**Authors:** Erik Domellöf, Anna-Maria Johansson, Aijaz Farooqi, Magnus Domellöf, Louise Rönnqvist

**Affiliations:** ^1^Department of Psychology, Umeå University, Umeå, Sweden; ^2^Division of Pediatrics, Department of Clinical Sciences, Umeå University, Umeå, Sweden

**Keywords:** children, preterm, gestational age, IQ, WISC-IV, CBCL 6–18, growth

## Abstract

This study aimed to investigate cognitive and behavioral outcomes in relation to gestational age (GA) in school-aged children born preterm (PT). Results from the Wechsler Intelligence Scale for Children, 4th edition (WISC-IV), and the Child Behavior Checklist (CBCL) were analyzed in 51 children (mean age: 7.8 years [range: 7.0–8.7]) born PT (mean GA: 31 weeks [range: 23–35]; birth weight, mean: 1,637 g [range: 404–2,962]) with the majority (96%) having no diagnosed cognitive, sensory, or motor impairments. The control group included 57 age-matched typically developing children (mean age: 7.9 years [range: 6.2–8.7]) born full-term (FT). Children born PT, extremely PT (GA < 28) in particular, showed significantly lower cognitive performance and higher behavioral problem scores compared with children born FT. GA was found to predict aspects of both cognitive functioning and behavioral problems within the PT group, with lower GA being related to both poorer cognitive outcomes and elevated affective and attention-deficit/hyperactivity problems. Global cognitive functioning did not independently predict aspects of behavioral outcomes. Findings demonstrate that, even in children born PT without severe perinatal and/or postnatal complications and receiving active perinatal care, a short gestation is an evident risk factor for long-term negative effects on mental health independent of cognitive functioning. Additional findings suggest that both reduced growth and lower parental educational level may contribute to increased risk for poorer cognitive and behavioral functioning in children born PT.

## Introduction

A large body of evidence on long-term impaired functionality in children born preterm (PT, < 37 weeks' gestation, GW) consistently suggest cognitive and behavioral difficulties as two major problem areas (1–8). Cognitive and behavioral deficits can be assumed originating from underlying neurological disruption associated with a PT birth (9). Neurodevelopmental problems are often more severe with, but not restricted to, increased immaturity at birth (3, 5, 10). As improvements in perinatal care from the late 1990s have led to increased survival rates of children born PT (11), more children are at risk for a disadvantageous development. It is widely recognized that increased knowledge of the long-term neurodevelopmental presentations of surviving children after active perinatal care is imperative to help tailor relevant and timed interventions. Outcomes from a recent meta-analysis and systematic review (12) describe strong relationships between gestational age (GA) and later cognitive abilities and academic performances in children born PT. Additionally, it was found that attention-deficit hyperactivity disorder (ADHD) was diagnosed twice as often in children born PT compared with those born full-term (FT) and that neurodevelopmental deficits in children born PT persist beyond primary school age (12). Yet, emerging neurodevelopmental problems in children born PT with no diagnosed impairment are difficult to predict with accuracy and may not be recognized until middle childhood (13). At this age, children born PT may display both poorer cognitive and educational achievements (1, 2, 14), more externalizing behavioral disturbances such as impulsivity and hyperactivity, and internalizing behaviors such as anxiety, depression, and social problems (1–3, 8, 15) than peers born term. Lowered cognitive performance has been associated with behavioral problems in school-aged children born PT, suggesting that the latter may be mediated by the former (3, 16, 17). Early school age is a sensitive period in terms of increased cognitive and socio-behavioral pressure, which may be a triggering factor for emerging difficulties in children born PT. Children with intellectual deficits at early school age have been reported displaying more externalizing behavioral problems than typically developing children, linked to new demands on sociability, autonomy, identity development, and adjustments to the learning context (18). However, an increased risk for behavioral problems independent of cognitive difficulties has also been noted in children born PT at school age (3, 8, 16). Studies of children born very PT or with very low birth weight (BW) and adolescents born extremely PT have shown consistency in patterns of behavioral problems, although estimates vary (4, 19–21). Still, there is no general agreement between studies whether behavioral problems in school-aged children born PT are of mainly externalizing or internalizing nature. This discrepancy is likely related to known differences in both cognitive and behavioral presentations depending on whether children have been born at an extremely, very, or moderately/late gestational age (10). Additional study to corroborate such proposed relations is however required.

Further, birth immaturity may also involve growth restriction in developing children, with potential impact on cognitive and behavioral functioning. Postnatal growth (i.e., weight, height, and body mass index, BMI) in children born PT has been associated with both short- and long-term neurodevelopmental outcome (22–25). A fast growth gain during infancy has been found related to better cognitive outcomes in later childhood, although it comes with a risk of developing obesity (25). Children born PT typically lag behind in growth compared with those born FT during development (26). There is a catch-up in growth at school age, although not necessarily to the level of FT peers (27). Growth attainment in children born PT at an early school age has previously been related to behavioral measures such as cognitive functioning and motor performance (23–25), also in relation to obesity (25), and small for gestational age (SGA) status (23). Still, the role of growth progress for behavioral functioning in developing children born PT is far from understood, and increased knowledge of growth-behavior associations is warranted.

Moreover, socioeconomic differences (i.e., parental educational accomplishment, occupation, and/or income) have been suggested related to both cognitive performance and brain development over the course of childhood and beyond (28, 29). In PT pediatric populations, low parental socioeconomic status in children born moderately PT has been found to associate with greater emotional and behavioral problems (30). A recent study of children born very PT compared with FT at 8–12 years found that children with two highly educated parents performed better than did children with just one highly educated parent on most measures of cognitive and behavioral functioning (31). Therefore, independently and/or in interaction, pre- and perinatal risk factors, GA, and parental socioeconomic status are seemingly important determinants that may influence cognitive performance and mental health in both children born PT and FT.

In the present study, we set out to explore cognitive and behavioral outcomes relative to GA in a sample of school-aged children born PT (GA 23–35) in the early 2000s compared with age-equivalent children born term. In order to add further to the field, we aimed to specifically investigate whether behavioral problems following a PT birth appear to be mainly externalizing or internalizing, and possible associations between such problems and cognitive functioning in relation to immaturity at birth. Regarding growth, we included an exploration of relations between parent-reported anthropometric measures and measures of cognitive and behavioral performance. Additionally, we investigated possible associations between parental education levels and children's cognitive performance and behavioral outcome scores.

## Materials and Methods

### Participants

**Figure 1:**
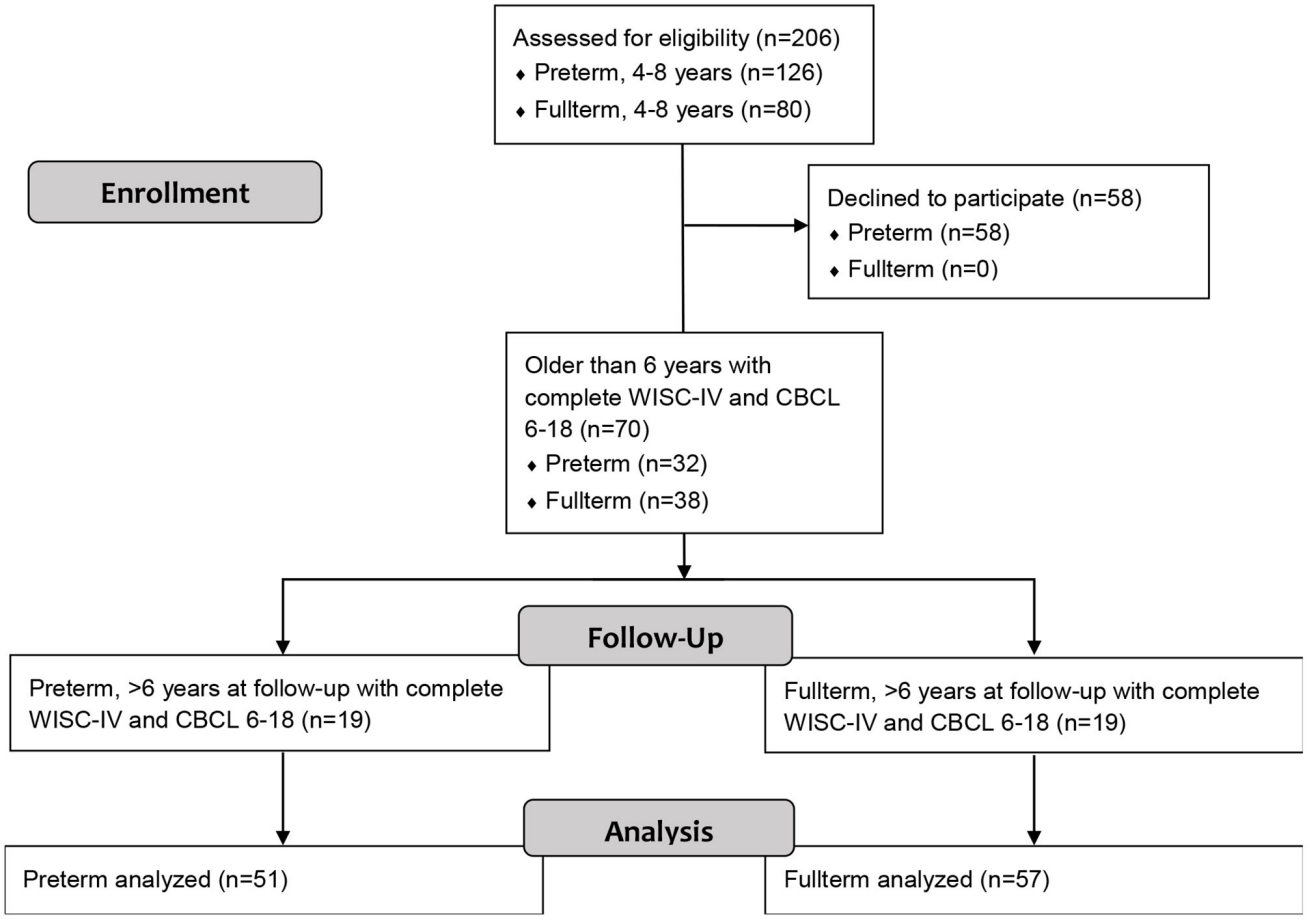
Flow diagram of the study enrollment process.

As part of a prospective multidisciplinary follow-up framework study, 126 children born PT between 2000 and 2005 aged 4–8 years were identified through birth records at the tertiary level care center of the Umeå University Hospital, Sweden, and contacted for participation (Figure 1). Of these, 68 children accepted (≤35 GW, range 22–35, M = 31.7, SD = 3.4, 30 girls). The children born PT who declined participation (46%) had a significantly higher GA (M = 33 GW, *p* < 0.01) than those who accepted. Thirty-two of the 68 participating children born PT were above 6 years of age, successfully completed the Wechsler Intelligence Scale for Children, 4th edition (WISC-IV, 6–16 years), and had a complete Child Behavior Checklist, 6–18 years (CBCL 6–18) provided by the parents. Identification of control children was made by selecting healthy at birth (according to ICD-10) children born term at the same hospital, of the same sex, and nearest in birth date (7 days) to the children born preterm. Following these criteria, a total of 80 healthy children born FT were recruited of which 38 (>6 years old) provided results from both the WISC-IV and CBCL 6–18. The youngest children, not qualified to be assessed with WISC-IV at the original testing session, were followed up 4 years later. This resulted in the possibility to add 19 children born PT and 19 control children with completed outcomes from the WISC-IV and CBCL 6–18 to the present study. Thus, the final sample consisted of 108 children (45 girls) at 6–8 years of age (M = 7.9, SD = 0.6), of which 51 (21 girls) had been born PT (≤35 GW), and 57 (24 girls) FT (≥38 GW). Two of the children born PT (two boys) had mild hemiplegic cerebral palsy (HCP), and 9 had been born SGA. Notably, all participating children born PT had received active perinatal care at the same highly specialized hospital, the majority (96%) did not have any diagnosed neurodevelopmental disorder when assessments were carried out, and all attended regular school. All participants born FT were healthy, typically developing children. The study was approved by the local ethical committee (registration nr 05–104 M) and was conducted in accordance with the Declaration of Helsinki. All parents and children gave informed consent for participation. Participant characteristics are given in Table 1.

**Table 1:** Participant characteristics.

**Variable**	**PT group (*N* = 51)**	**FT group (*N* = 57)**
Maternal education (years), M (SD [range])	14.3 (2.6 [9–22])	14.6 (2.4 [11–25])
SUN	46.0 (10.9 [20–64])	47.3 (9.3 [32–60])
Paternal education (years), M (SD [range])	13.5 (2.6 [9–21])	14.0 (3.1 [9–20])
SUN	41.9 (11.0 [20–64])	43.2 (11.7 [20–64])
Age at assessment, M (SD [range])	7.8 (0.6 [7.0–8.7])	7.9 [0.6 (6.2–8.7])
Birth weight (grams), M (SD [range])^***^	1,637 (690 [404–2,962])	3,754 (401 [3,130–4,790])
Gestational age (weeks), M (SD [range])^***^	31.1 (3.5 [23–35])	40.3 (0.9 [38–42])
Sex (% male)	59%	58%
Height at assessment (cm), M (SD [range]), N_PT_ = 47, N_FT_ = 51^*^	128.0 (7.7 [109–151])	131.2 (6.5 [120–146])
Weight at assessment (kg), M (SD [range]), N_PT_ = 47, N_FT_ = 50^*^	26.2 (6.3 [17–50])	29.7 [7.0 [21–50]]
ISO-BMI at assessment (kg/m^2^), M (SD [range]), N_PT_ = 47, N_FT_ = 50^**^	15.7 (2.3 [13–23])	17.1 (2.7 [13–25])

*PT, preterm; FT, full-term; M, mean; SD, standard deviation; SUN, Swedish Classification of Education; ISO-BMI, age- and sex-adjusted body mass index*.

* *p < 0.05*;

** *p < 0.01*;

*** *p < 0.001 as tested by one-way ANOVA*.

### Measures and Procedures

#### Cognitive Assessment

Assessment of cognitive functioning was made by the Swedish version of the Wechsler Intelligence Scale for Children, 4th edition (WISC-IV), covering the following domains: verbal comprehension (verbal comprehension index, VCI), perceptual (non-verbal) reasoning (perceptual reasoning index, PRI), working memory (WM), and processing speed (PS), jointly generating a full-scale intelligence quotient (FSIQ). Assessments were carried out in a dedicated testing room by a neuropsychologist (first author, ED) and two trained advanced-level clinical psychologist students (supervised by ED).

#### Behavioral Assessment

Assessment of behavioral functioning was made by the Child Behavior Checklist, 6–18 years (CBCL 6–18, Swedish version), a standardized instrument providing parental report of emotional, social, and behavioral problems in children as observed within the last 6 months (32). The CBCL is composed of 113 items on a three-point Likert scale (0 = “not true”, 1 = “somewhat or sometimes true,” and 2 = “very true or often true”) generating a total problem score based on the aggregated scores for the subscales Anxiety/depression, Withdrawal, Somatic complaints, Social problems, Thought problems, Attention problems, Rule-breaking, and Aggressive behavior. Based on these subscales, the CBCL provides two broadband factors of behavioral problems: internalizing (Anxiety/depression, Withdrawal, and Somatic complaints) and externalizing (Attention problems, Aggressive behavior, and Rule-breaking). In addition, six Diagnostic and Statistical Manual of Mental Disorders (DSM)-oriented syndrome scales can be extracted: Affective problems, Anxiety problems, Somatic problems, Attention deficit/hyperactivity (ADH) problems, Oppositional defiant problems, and Conduct problems. Summed raw scores are converted to age- and sex-specific normalized T scores. T scores for total, internalizing, and externalizing behavioral problems >63 denote clinical significance (borderline range 60–63). For the syndrome scales, T scores ≥70 indicate clinical significance (borderline range 55–69).

In addition, information about the child's physical activity from the CBCL background data was extracted, calculated as the Activities scale of CBCL (26). The Activities scale consists of parental ratings of approximately how much time the child spends and how skillfully they perform each specified sport compared to other children of the same age. Ratings are made on a scale from 0 to 2 (0 = “below than average,” 1 = “average,” and 2 = “above average”).

#### Parental Level of Education

To evaluate parental level of education, the Swedish Standard Classification of Education (SUN) was used (33). SUN is a classification system that aggregates education into larger groups. The system is based on two hierarchical modules, Level of education, and Fields of Education. Level of Education module is defined by which educational level the person has been studying at, for how many years in total, and if the education was vocational or of general type. It also indicates a person's main work orientation and specialization. Fields of Education module is defined by rough and specified measurements and adjusted to the International Standard Classification of Education (ISCED 97).

### Statistical Analyses

Analyses of variance (ANOVA) were used to test the effect of sex (boy, girl) and group (FT, PT) on the behavioral and cognitive outcome measures. Analyses of potential subgroup differences in relation to the degree of prematurity (FT, moderately PT [MPT, GA = 33–35 weeks], very PT [VPT, GA 28–32 weeks], extremely PT [EPT, GA < 28 weeks]) were performed. For these analyses, partial eta-squared ($\eta_{\text{p}}^{2}$) is reported as a measure of effect size, and the Tukey HSD test was employed for *post hoc* analyses. Correlation analyses by Pearson's r were used to investigate associations between the factors of interest (BW, GA, FSIQ, WISC-IV outcomes, CBCL 6–18 behavioral problems, CBCL 6–18 syndrome scales). As GA and BW correlated strongly (*r* =0.95), only GA was included in the analyses. Multiple linear regression analyses were used to predict CBCL 6–18 outcomes from GA and FSIQ and WISC-IV outcomes from GA and CBCL 6–18 total problem score. Additionally, within-group (FT/PT) correlations were made between anthropometric measures (height, weight, age-, and sex-adjusted BMI [ISO-BMI]) and cognitive and behavioral outcomes and between parental education level and FSIQ and CBCL 6–18 main behavioral problem and physical activity scores. Correction for multiple comparisons was not applied due to the risk for excessive power reduction when outcome variables are related (34). The alpha level was set at *p* < 0.05 for all analyses.

## Results

Note that excluding the two participating children with HCP (both born VPT) did not significantly change the results presented in this section.

### Group Differences

#### Cognitive Performance

Separate 2 (sex: boy, girl) by 2 (group: PT, FT) ANOVAs for the WISC-IV index outcomes revealed no main effect of sex and no significant sex by group interaction. However, a main effect of group was found for FSIQ, VCI, PRI, and WM, but not for PS (Table 2). The group effects were characterized by a lower general cognitive score for children born PT compared with FT controls.

**Table 2:** Mean composite scores for the WISC-IV indices presented by group (PT, FT) and sex (girl, boy).

	**PT (*n* = 51, 21 girls)**	**FT (*n* = 57, 24 girls)**	**ANOVA**	**Effect size**
**WISC-IV measure**	**Mean (SD)**	**Mean (SD)**	***F* (*p*-value)**	**$\eta_{\text{p}}^{2}$**
Verbal comprehension index (VCI)	96.0 (10.1)^*^	102.8 (10.3)	10.21 (**0.002**)	0.09
Girls	99.3 (10.7)	102.8 (11.0)		
Boys	93.7 (9.2)	102.8 (9.9)		
Perceptual reasoning index (PRI)	101.1 (14.2)^*^	109.5 (11.3)	11.43 (**0.001**)	0.10
Girls	100.2 (12.3)	108.8 (12.9)		
Boys	101.7 (15.5)	110.1 (10.1)		
Working memory index (WM)	87.3 (12.0)^*^	92.6 (11.2)	5.63 (**0.019**)	0.05
Girls	87.4 (9.4)	93.8 (12.3)		
Boys	87.2 (13.6)	91.7 (10.4)		
Processing speed index (PS)	95.3 (14.0)	97.4 (12.2)	0.46 (0.501)	0.00
Girls	98.6 (11.6)	98.0 (14.0)		
Boys	93.0 (15.2)	97.0 (10.9)		
Full-scale intelligence quotient (FSIQ)	94.4 (11.1)^*^	102.6 (10.3)	14.39 (**0.000**)	0.12
Girls	96.1 (7.9)	103.0 (11.0)		
Boys	93.2 (12.9)	102.2 (10.0)		

*WISC-IV, Wechsler Intelligence Scale for Children, 4^th^ edition; PT, preterm; FT, full-term. Significant main effect of group is indicated in bold*.

* *Significant difference between the PT and the FT group*.

As shown in Table 3, one-way ANOVAs including a subdivision of the PT group depending on degree of prematurity (EPT, VPT, MPT) confirmed main effects of group for FSIQ, VCI, PRI, and WM. *Post hoc* testing further revealed evident differences between the EPT and FT group (Table 3), suggesting a relation between GA and cognitive function particularly accentuated for children born early in gestation.

**Table 3:** Mean composite scores for the WISC-IV indices presented by subgroup.

	**EPT (*n* = 13)**	**VPT (*n* = 16)**	**MPT (*n* = 22)**	**FT (*n* = 57)**	**ANOVA**	**Effect size**
**WISC-IV measure**	**Mean (SD)**	**Mean (SD)**	**Mean (SD)**	**Mean (SD)**	***F* (*p*-value)**	**$\eta_{\text{p}}^{2}$**
Verbal comprehension index	92.5 (8.8)^*^	95.9 (10.8)	98.1 (10.2)	102.8 (10.3)	4.87 (**0.003**)	0.12
Perceptual reasoning index	93.6 (9.9)^*^	103.8 (14.6)	103.5 (14.9)	109.5 (11.3)	6.21 (**0.001**)	0.15
Working memory index	81.6 (8.3)^*^	90.1 (9.1)	88.6 (14.6)	92.6 (11.2)	3.39 (**0.021**)	0.09
Processing speed index	93.2 (17.4)	92.8 (11.8)	98.4 (13.3)	97.4 (12.2)	0.96 (0.417)	0.03
Full-scale intelligence quotient (IQ)	88.2 (9.9)^*^	95.6 (9.9)	97.2 (11.6)	102.6 (10.3)	7.58 (**0.000**)	0.18

*WISC-IV, Wechsler Intelligence Scale for Children, 4^th^ edition; EPT, extremely preterm; VPT, very preterm; MPT, moderately preterm; FT, full-term. Significant main effect of group is indicated in bold*.

* *Significant difference between the EPT and the FT group*.

#### Behavioral Outcomes

Corresponding analyses for the CBCL 6–18 T score outcomes yielded no main effect of sex or group for either the main behavioral problem scores (total problems, internalizing problems, externalizing problems) or the DSM-oriented syndrome scales. Complementary analyses at subgroup level, however, revealed a main effect of group for the respective syndrome scales Affective problems, ADH problems, and Conduct problems, where the children born EPT displayed significantly higher problem scores than the FT, MPT, and VPT groups did, respectively (Table 4).

**Table 4:** Mean CBCL 6–18 T score outcomes presented by subgroup.

	**EPT (*n* = 13)**	**VPT (*n* = 16)**	**MPT (*n* = 22)**	**FT (*n* = 57)**	**ANOVA**	**Effect size**
**CBCL factor**	**Mean (SD)**	**Mean (SD)**	**Mean (SD)**	**Mean (SD)**	**F (*p*-value)**	**$\eta_{\text{p}}^{2}$**
Total problem score	50.8 (14.2)	47.4 (6.7)	43.5 (8.9)	47.4 (7.0)	2.15 (0.099)	0.06
Internalizing problems	52.2 (9.8)	50.5 (7.5)	46.8 (8.2)	49.1 (7.3)	1.48 (0.225)	0.04
Externalizing problems	50.6 (12.4)	46.5 (9.3)	44.1 (7.7)	46.8 (8.2)	1.46 (0.229)	0.04
Affective problems	57.6 (8.1)^*^	51.1 (1.9)	50.3 (0.5)	51.6 (2.8)	13.58 (**0.000**)	0.28
Anxiety problems	52.5 (3.8)	52.9 (5.3)	51.6 (3.4)	52.0 (2.9)	0.54 (0.659)	0.02
Somatic problems	55.2 (7.3)	54.9 (6.4)	52.8 (5.7)	54.5 (5.9)	0.63 (0.597)	0.02
ADH problems	56.4 (8.2)^*^	52.4 (2.9)	51.4 (1.9)	51.8 (2.9)	6.02 (**0.001**)	0.15
Oppositional defiant problems	56.5 (7.7)	54.4 (7.5)	52.2 (4.6)	53.4 (4.6)	1.75 (0.161)	0.05
Conduct problems	55.5 (6.7)^*^	51.6 (4.3)	51.8 (3.6)	51.9 (2.9)	3.48 (**0.019**)	0.09

*CBCL 6–18, Child Behavior Checklist, 6–18 years; EPT, extremely preterm; VPT, very preterm; MPT, moderately preterm; FT, full-term; ADH, attention deficit/hyperactivity. Significant main effect of group is indicated in bold*.

* *Significant difference between the EPT and the respective VPT, MPT and FT group*.

### Relations Between Outcome Data

No significant associations were found within the FT control group. Thus, reported results in this section only apply to the PT group.

#### Correlation Analyses

**Figure 2:**
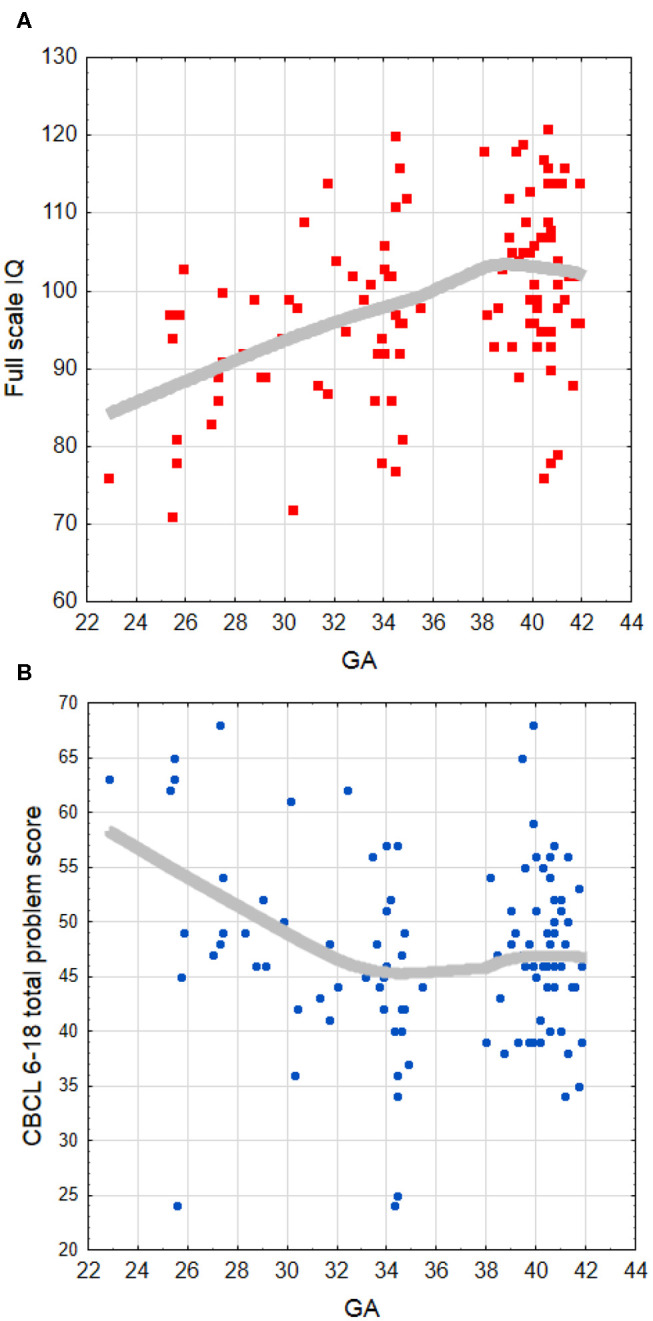
Locally weighted (LOESS curve) fit to the scatter plot of the association between **(A)** full-scale IQ and GA, and **(B)** CBCL 6–18 total problem score and GA. The LOESS fitting indicates that relations are not truly linear, with a saturation effect appearing approximately after 34–35 weeks of GA. GA, gestational age (weeks).

Within the whole group of children born PT (N = 51), significant correlations were found between GA and FSIQ (*r* =0.38, *p* < 0.01; Figure 2A), VCI (*r* =0.31, *p* < 0.05), and PRI (*r* = 0.28, *p* < 0.05), suggesting that a shorter gestation is related to poorer cognitive functioning. Regarding CBCL 6–18 outcomes, GA was found evidently negatively associated with total problems (*r* = −0.31, *p* < 0.05; Figure 2B), internalizing problems (*r* = −0.30, *p* < 0.05), affective problems (*r* = −0.51, *p* < 0.001), ADH problems (*r* = – 0.44, *p* < 0.01), and conduct problems (*r* = −0.30, *p* < 0.05), indicating more behavioral problems with lower GA. One significant association was also found between the WISC-IV and CBCL 6–18 outcomes in terms of lower WM scores being related to higher scores linked to problems with attention/hyperactivity (*r* = −0.35, *p* < 0.05).

#### Regression Analyses

Multiple linear regressions were calculated within the preterm group to predict CBCL 6–18 outcomes based on GA and FSIQ, and WISC-IV outcomes based on GA and CBCL 6–18 total problem scores. Significant regression equations were found for Affective problems, *F*_(2, 48)_ = 8.61, *p* < 0.001, with an *R*^2^ of 0.264, ADH problems, *F*_(2, 48)_ = 6.23, *p* < 0.01, with an *R*^2^ of 0.206, and FSIQ, *F*_(2, 48)_ = 4.12, *p* < 0.05, with an *R*^2^ of 0.147. Regression equations for VCI, *F*_(2, 48)_ = 2.89, *p* < 0.07, with an *R*^2^ of 0.107, and PRI, *F*_(2, 48)_ = 3.00, *p* < 0.06, with an *R*^2^ of 0.111, just failed to reach statistical significance. As seen in Table 5, GA was a significant predictor of both behavioral problems (Affective and ADH) and lowered cognitive functioning (FSIQ, VCI, and PRI). FSIQ and CBCL 6–18 total problem score, separately, added no unique variance to the respective predictions of behavioral problems or cognitive functioning. Thus, in the present sample, there was an evident interrelation between degree of prematurity and both cognitive and syndrome-related behavioral problems. However, FSIQ did not independently predict aspects of behavioral problems, and CBCL 6–18 total problem score did not independently predict aspects of cognitive functioning.

**Table 5:** Outcomes from multiple linear regression analyses predicting respective CBCL 6–18 and WISC-IV outcomes from gestational age, general cognitive functioning, and total behavioral problem score for the children born preterm.

	**PT group (*****N*** **= 51)**				
**CBCL 6–18 factors**	**Predictor**	**β**	**β_**SD**_**	***t*_**(48)**_**	***p*-value**
Total problem score (TPS)	GA	–0.848	0.430	−1.970	0.055
	FSIQ	–0.037	0.136	–0.275	0.785
Internalizing problems	GA	–0.686	0.361	–1.899	0.064
	FSIQ	–0.033	0.114	–0.290	0.773
Externalizing problems	GA	–0.783	0.416	–1.882	0.066
	FSIQ	0.031	0.132	0.238	0.813
Affective problems	GA	−0.731	0.196	−3.733	**0.001**
	FSIQ	−0.016	0.062	−0.254	0.801
Anxiety problems	GA	−0.070	0.182	−0.386	0.701
	FSIQ	−0.037	0.057	−0.646	0.521
Somatic problems	GA	−0.454	0.272	−1.668	0.102
	FSIQ	0.072	0.086	0.836	0.407
ADH problems	GA	−0.570	0.194	−2.933	**0.005**
	FSIQ	−0.043	0.061	−0.697	0.489
Oppositional defiant problems	GA	−0.528	0.280	−1.887	0.065
	FSIQ	0.019	0.088	0.211	0.834
Conduct problems	GA	−0.365	0.212	−1.722	0.091
	FSIQ	−0.057	0.067	−0.853	0.398
**WISC-IV indices**	**Predictor**	***β***	***β***_**SD**_	***t***_**(48)**_	***p*****-value**
FSIQ	GA	1.167	0.443	2.634	**0.011**
	TPS	−0.042	0.153	−0.275	0.785
VCI	GA	0.988	0.412	2.400	**0.020**
	TPS	0.121	0.142	0.850	0.399
PRI	GA	1.365	0.575	2.374	**0.022**
	TPS	0.258	0.198	1.300	0.200
WM	GA	0.576	0.491	1.171	0.247
	TPS	−0.233	0.169	−1.377	0.175
PS	GA	0.549	0.577	0.951	0.346
	TPS	−0.303	0.199	−1.526	0.134

*PT, preterm; CBCL 6–18, Child Behavior Checklist, 6–18 years; TPS, total problem score; GA, gestational age; ADH, attention deficit/hyperactivity; FSIQ, full-scale intelligence quotient; VCI, verbal comprehension index; PRI, perceptual reasoning index; WM, working memory; PS, processing speed. Significant outcomes are indicated in bold*.

#### Associations Between Anthropometric Measures and Child Outcome Data

Table 1 displays the mean and SD for height, weight, and ISO-BMI for the respective group (PT vs. FT) at age of assessment. As shown, the FT group was significantly taller and heavier, with an evidently higher ISO-BMI than the PT group. According to widely accepted ISO-BMI cutoff points for overweight and obesity (35), 4 children born FT were overweight (8%) and 6 obese (12%). In the PT group, 5 children were overweight (11%) and 1 was obese (2%). No significant associations between anthropometric measures and cognitive/behavioral outcome scores were found within the FT group. As shown in Table 6, however, height and weight, but not ISO-BMI, were significantly associated with both behavioral and cognitive outcome scores within the PT group. More specifically, height was found positively correlated with all WISC-IV indices except VCI and negatively with CBCL 6–18 behavioral problems (Total problem score, Affective, and ADH). Weight was found positively correlated with FSIQ and WM and negatively with affective problems. Notably, within the PT group, there was no significant difference in any measure between children born SGA and those born appropriate for GA.

**Table 6:** Within group correlations between child outcome scores regarding CBCL 6–18 factors, WISC-IC indices, and parent-reported anthropometric measures at assessment age for the children born preterm.

	**PT group (*****N*** = 47^*^)		
**CBCL 6–18 factors**	**Height**	**Weight**	**ISO-BMI**
Total problem score	***r*** **= −0.322**, ***p*** **= 0.048**	*r* = −0.240, *p* = 0.15	*r* = −0.174, *p* = 0.30
Internalizing problems	*r* = −0.245, *p* = 0.14	*r* = −0.176, *p* = 0.29	*r* = −0.166, *p* = 0.32
Externalizing problems	*r* = −0.311, *p* = 0.057	*r* = −0.265, *p* = 0.11	*r* = −0.191, *p* = 0.25
Affective problems	***r*** **= −0.323**, ***p*** **= 0.048**	***r*** **= −0.348**, ***p*** **=0.032**	*r* = −0.295, *p* = 0.072
Anxiety problems	*r* = −0.000, *p* = 0.99	*r* = −0.063, *p* = 0.71	*r* = −0.191, *p* = 0.25
Somatic problems	*r* = − 0.191, *p* = 0.25	*r* = −0.167, *p* = 0.32	*r* = −0.177, *p* = 0.29
ADH problems	***r*** **= −0.390**, ***p*** **=0.015**	*r* = −0.300, *p* = 0.067	*r* = −0.151, *p* = 0.37
Oppositional defiant problems	*r* = −0.168, *p* = 0.31	*r* = −0.100, *p* =0.55	*r* = −0.013, *p* = 0.94
Conduct problems	*r* = −0.281, *p* =0.087	*r* = −0.207, *p* = 0.21	*r* = −0.109, *p* = 0.51
**WISC–IV indices**	**Height**	**Weight**	**ISO-BMI**
FSIQ	***r*** **= 0.432**, ***p*** **= 0.003**	***r*** **= 0.303**, ***p*** **= 0.041**	*r* = 0.115, *p* = 0.45
VCI	*r* = −0.002, *p* = 0.99	*r* = 0.002, *p* = 0.99	*r* = 0.013, *p* = 0.93
PRI	***r*** **= 0.393**, ***p*** **= 0.007**	*r* = 0.247, *p* = 0.098	*r* = 0.007, *p* =0.96
WM	***r*** **= 0.466**, ***p*** **= 0.001**	***r*** **= 0.394**, ***p*** **= 0.007**	*r* = 0.205, *p* =0.17
PS	***r*** **= 0.346**, ***p*** **= 0.018**	*r* = 0.156, *p* = 0.30	*r* = –0.018, *p* = 0.90

* *Only those for whom anthropometric measures were available for both height and weight were included in the analyses. PT, preterm; CBCL 6–18, Child Behavior Checklist, 6–18 years; TPS, total problem score; ADH, attention deficit/hyperactivity; FSIQ, full-scale intelligence quotient; VCI, verbal comprehension index; PRI, perceptual reasoning index; WM, working memory; PS, processing speed. Significant correlations (p < 0.05) are indicated in bold*.

#### Associations Between Parental Education Level and Child Outcome Data

No significant group difference regarding education classification between parents of children born FT and PT was found (number of years of education: *F*_(2, 110)_ = 1.66, *p* = 0.196; SUN: *F*_(2, 110)_ = 1.25, *p* = 0.292). The parental SUN classification and number of years of education (independent of child GA) were strongly positively correlated for both mothers, *N* = 96, *r* = 0.89, *p* < 0.0001, and fathers, *N* = 96, *r* = 0.93, *p* < 0.0001. Thus, only SUN classification was used for the correlation analyses between parental education level and child outcome data. As shown in Table 7, significant negative associations between main behavior problem scores and paternal SUN classification were found within the PT group, indicating a link between both elevated internalizing and externalizing problems in the children and lower educational levels in the fathers. Further, significant positive correlations were found between physical activity and FSIQ in the children born PT and maternal SUN classification, suggesting that more physical activity and higher IQ scores were associated with more highly educated mothers. For the FT children, no significant associations between behavior problem scores and parental SUN classification were found. However, positive correlations between FSIQ in children born FT and both paternal and maternal SUN classification were found, indicating that higher IQ scores were associated with more highly educated parents.

**Table 7:** Within group (preterm/full-term) correlations between child outcome scores regarding internalizing/externalizing behavioral problem, physical activity, full-scale IQ, and outcomes from the Swedish Classification of Education (SUN) for the parents.

**SUN**	PT group (*N* = 43^*^)	FT group (*N* = 53^*^)
**INTERNALIZING PROBLEMS**		
Mother	*r* = −0.118, *p* = 0.45	*r* = 0.048, *p* = 0.72
Father	*r* **= −0.383**, ***p*** **= 0.01**	*r* = 0.086, *p* = 0.73
**EXTERNALIZING PROBLEMS**		
Mother	*r* = −0.133, *p* = 0.39	*r* = 0.158, *p* = 0.28
Father	***r*** **= −0.361**, ***p*** **= 0.02**	*r* = 0.086, *p* = 0.54
**PHYSICAL ACTIVITY (AMOUNT + SKILLFULNESS)**		
Mother	***r*** **= 0.310**, ***p*** **= 0.04**	*r* = 0.238, *p* = 0.09
Father	*r* = 0.232, *p* = 0.13	*r* = 0.045, *p* = 0.75
**FULL–SCALE IQ**		
Mother	***r*** **= 0.319**, ***p*** **= 0.03**	***r*** **= 0.351**, ***p*** **= 0.01**
Father	*r* = 0.136, *p* = 0.38	***r*** **= 0.288**, ***p*** **= 0.04**

* *Only those for whom SUN classification was available for both parents were included in the analyses. PT, preterm; FT, full-term; IQ, intelligence quotient. Significant correlations (p < 0.05) are indicated in bold*.

## Discussion

In the present study, outcomes from WISC-IV and CBCL 6–18 were compared between, and associated within, children born PT and FT controls at early school age. The children born PT, particularly those born at early GA, displayed lowered cognitive performance and increased syndrome-related behavioral problems, of mainly internalizing nature, compared with children born FT. In keeping, GA was found to predict aspects of both cognitive functioning and behavioral problems within the PT group in terms of short gestation being related to both poorer cognitive and behavioral outcomes in the children born PT. No unique contribution of cognitive functioning to behavioral problems or vice versa was however found. Consequently, this study provides additional evidence of increased risk for mainly independent cognitive and syndrome-related behavioral problems in early school-aged children born at low GA in the early 2000s.

It should be noted that the children born PT in the present study displayed cognitive performance mainly within the average range and behavioral problems below clinical significance. Still, in keeping with previous studies on cognitive and behavioral functioning in school-aged children born at low GA after improvements in neonatal care (5, 15), the children born PT, EPT mainly, displayed poorer outcomes than FT controls.

Regarding global cognitive functioning, children born PT generally displayed lowered FSIQ than FT controls, with the EPT group mean FSIQ within the low average range. The prominent difference in IQ score between children born EPT and FT controls (mean 14 points) is in keeping with meta-analytic findings (2, 6, 36, 37) and indicative of a persistent long-term effect of EPT birth on cognitive functioning with relevance for academic performance. Notably, the WM index score for the EPT group was specifically low (M = 81.6, approaching borderline range), suggesting an increased risk for particular problems with executive functioning. Recent research has also shown that poor attention and working memory, which are important elements of executive functioning, strongly predict academic achievement in children born EPT with or without significant intellectual disabilities (38–40).

As for parent-reported behavioral problems, evident differences between the PT and FT groups were restricted to the DSM-oriented scales Affective problems, ADH problems, and Conduct problems, driven by augmented scores in the EPT group. This finding could cautiously be interpreted as an increased risk for various forms of psychiatric disorders in the lower gestational age range of preterm birth. Similar to cognitive functioning, these problem areas also mirror those observed in previous research (1–3, 5, 15), in particular with regard to inattention–hyperactivity and emotional problems. The moderate negative correlation between lower WM scores and ADH problems within the PT group, also considering the poor WM outcome for the EPT group, further suggests a particular risk for ADHD in children born at the lower GA range. This interpretation is also in keeping with recent overviews concerning children born EPT after the 1990s (5, 15).

Moreover, studies have reported elevated total, internalizing, and/or externalizing problem scores in children born PT compared with FT peers (2) and that higher levels of internalizing difficulties may be a particular and long-lasting characteristic in children born at lower GA in the 1990s−2000s (4, 8, 14, 15, 41). Recently, an increased risk of externalizing problems at 7 years of age in infants with marginally low birth weight (of which most were moderately/late preterm) has been described—a risk that may be minimized by preventing iron deficiency (42). We have no individual data of iron intake in the current study, but iron supplementation was generally recommended for infants born PT with birth weight <2,500 g. No effect of group on total problem score and no specific pattern regarding internalizing or externalizing problems in the PT group could be discerned in the present study. However, evident correlations between lower GA and increased total problem and internalizing problem scores were found, suggesting mainly internalizing problems in children born at lower GA in the present sample. In accordance, a strong association was found between GA and Affective problems, mainly characterized by introversion.

Significant associations were also evident between GA and WISC-IV and CBCL 6–18 outcomes, respectively. Consistent with reports of increased functional problems with decreased GA in children born PT (5), these findings further strengthen the notion that GA (and/or BW) remains an important predictor of cognitive and behavioral problems in children receiving active perinatal care. Here, these problems were specified to global cognitive functioning, verbal comprehension, and non-verbal reasoning in the cognitive domain and affective and attention/hyperactivity symptoms in the behavioral domain. Several additional near-significant findings were however revealed for the behavioral outcomes (Table 4), suggesting that more significant associations concerning risk for psychological health problems with increased birth immaturity could have been revealed if the study sample had been larger.

In contrast to previous findings (3, 16, 17), global cognitive functioning did not evidently predict behavioral outcomes in the present sample. This inconsistency may be due to between-study differences such as study sample size and participant age. An alternative explanation is that the perinatal care given to these children in a specialized Swedish University hospital setting has been successful in optimizing developmental progress so that this association is reduced at early school age (11). ADHD-related attention difficulties have been suggested related to regulation problems underlying lowered cognitive functioning in children born MPT at early school age (41). In our sample, however, total behavioral problem score was not a significant predictor of cognitive functioning. Still, it is possible that the pattern of difficulties mainly reflecting the internalizing/attention domain embedded in the total behavioral problem score observed in the present study may have exerted a greater impact on cognitive outcome in a larger population.

The low WM index score obtained for the FT group was somewhat unexpected. However, recent data from a national Swedish study (EXPRESS) suggested that the Swedish version of WISC-IV may underestimate the WM scores of FT controls at 6.5 years of age (43). We nevertheless believe that the differences in WISC-IV outcomes between children born PT and FT are valid despite the low WM index scores for both the EPT subgroup and controls.

In keeping with older studies investigating growth status in school-aged children born PT (23, 24), we found evident deficits in growth (height, weight, and ISO-BMI) in our sample of children born PT compared with FT peers. Thus, despite continued advances in perinatal care, and independent of obesity and SGA status, a risk for growth restriction seems to remain. Accordingly, it appears of importance to continue to monitor long-term postnatal growth status following a PT birth. This statement is corroborated by findings of significant associations between reduced height and weight and lower cognitive scores and stronger indications of behavioral problems in the PT group. The relations between growth data and cognitive functioning are similar to previous reports (23, 24). Less attention has been given to associations between growth data and psychological health problems. Casey and colleagues (23) did not find any differences in health behavior status (“CBCL total,” “General Health Survey/Mental health”) at 8 years between children born PT who had failed to thrive and comparison groups. In contrast, we found that anthropometric measures (height in particular) correlated significantly with CBCL 6–18 total problem scores and the DSM-oriented scales Affective problems and ADH problems. Further studies are warranted to continue investigating these suggested links. The lack of impact of ISO-BMI and SGA in our sample is consistent with previous studies (23, 25). Here, height is tentatively proposed as an important factor in relation to neurodevelopmental outcome in mainly able children born PT at school age.

In line with recent reports of socioeconomic disparity as a source of variation in children's cognitive development (28–30), associations between parental education level and global cognitive functioning in the participating children were also revealed in this study. These relationships were more prominent in the children born FT and seemingly more strongly associated with maternal than paternal educational attainment and occupation. Additionally, we found negative associations between elevated behavioral problems and lower paternal education level within the PT group. This outcome is in keeping with previous findings of children with one highly educated parent performing poorer than children with two highly educated parents (31). Consequently, parental educational level may be an important factor to include in studies of cognitive performance and development in children born PT and in relation to behavioral and mental problems. It should be noted that unrestrained predisposing elements such as pre- and perinatal risk factors, biological markers/brain morphology and development, parental stress factors, and even iron deficiency may have acted as confounders in the described relation between parental education level and child outcomes. Still, the findings indicate that socioeconomic factors may be especially critical among the most PT-born and disadvantaged children.

In conclusion, the present study adds support for a continued risk for increased cognitive and mental health problems with lower GA in apparently typically developing school-aged children born PT, with possible relations to both growth attainment and parental education level. Even though care and survival of infants born PT have increased significantly during the last 15–20 years, there is still an evident negative correlation between gestational age at birth and cognitive/behavioral function at school age also in more recent cohorts (e.g., 43). It is thus not clear if improved care will lead to better neurodevelopmental outcomes and not just to improved survival with similar outcomes. Obvious limitations of this study include a limited study sample, a relatively low participation percentage, and no GA group between 35 and 38 weeks. Thus, results should be interpreted taking into account these restrictions. Notably, however, given that all participants were essentially able, the study population may be considered as relatively homogenous and thus allowing for a smaller sample size. Further, even in the relatively small sample studied, with a limited number of high-risk children born EPT in the PT group, findings are strikingly similar to recent reports of cognitive and behavioral functioning in children born at lower GA. Awareness of this risk in clinical, family, and school settings is of importance to support psychosocial developmental progress and well-being in these children. Of particular concern is the risk for affective and attentional problems independent of apparent cognitive difficulties in children at early school age born at low GA. This calls for continued development of relevant intervention and support paradigms, tailored to degree of prematurity and individual strengths and weaknesses. In this endeavor, the underlying neurological base for cognitive and behavioral functioning in developing children born PT ought to be considered to further the understanding of the relation between GA and neurobehavioral presentation (44, 45).

## Data Availability Statement

The datasets generated for this study are available on request to the corresponding author.

## Ethics Statement

The studies involving human participants were reviewed and approved by Umeå regional ethical committee, Umeå University. Written informed consent to participate in this study was provided by the participants' legal guardian/next of kin.

## Author Contributions

ED performed the statistical analyses and drafted the initial manuscript. All authors contributed in conceptualizing and designing the study, took part in the data collection, critically reviewed the manuscript, and approved the final manuscript as submitted.

## Conflict of Interest

The authors declare that the research was conducted in the absence of any commercial or financial relationships that could be construed as a potential conflict of interest.

## References

[1] Aarnoudse-Moens CSH Weisglas-Kuperus N van Goudoever JB Oosterlaan J. Meta-analysis of neurobehavioral outcomes in very preterm and/or very low birth weight children. Pediatrics. (2009) 124:717–28. 10.1542/peds.2008-2816 19651588

[2] Bhutta A Cleves MA Casey PH Cradock MM Anand KJS. Cognitive and behavioral outcomes of school-aged children who were born preterm: a meta-analysis. JAMA. (2002) 288:728–37. 10.1001/jama.288.6.728 12169077

[3] Delobel-Ayoub M Arnaud C White-Koning M Casper C Pierrat V Garel M. Behavioral problems and cognitive performance at 5 years of age after very preterm birth: the EPIPAGE study. Pediatrics. (2009) 123:1485–92. 10.1542/peds.2008-1216 19482758

[4] Farooqi A Hägglöf B Gothefors L Sedin G Serenius F. Mental health and social competencies in 10- to 12-year-old children born at 23-25 weeks of gestation in the 1990s: a Swedish national prospective follow-up study. Pediatrics. (2007) 120:118–33. 10.1542/peds.2006-2988 17606569

[5] Hutchinson EA De Luca CR Doyle LW Roberts G Anderson PJ. School-age outcomes of extremely preterm or extremely low birth weight children. Pediatrics. (2013) 131:e1053–61. 10.1542/peds.2012-2311 23509167

[6] Kerr-Wilson CO Mackay DF Smith GCS Pell JP. Meta-analysis of the association between preterm delivery and intelligence. J Public Health. (2012) 34:209–16. 10.1093/pubmed/fdr024 21393308

[7] Linsell L Malouf R Morris J Kurinczuk JJ Marlow N. Prognostic factors for poor cognitive development in children born very preterm or with very low birth weight. A systematic review. JAMA Pediatrics. (2015) 169:1162–72. 10.1001/jamapediatrics.2015.2175 26457641 PMC5122448

[8] Samuelsson M Holsti A Adamsson M Serenius F Hägglöf B Farooqi A. Behavioral patterns in adolescents born at 23 to 25 weeks of gestation. Pediatrics. (2017) 140:e20170199. 10.1542/peds.2017-0199 28642374

[9] Volpe JJ. Neurobiology of periventricular leukomalacia in the premature infant. Pediatr Res. (2001) 50:553–62. 10.1203/00006450-200111000-00003 11641446

[10] De Jong M Verhoeven M van Baar AL. School outcome, cognitive functioning, and behaviour problems in moderate and late preterm children and adults: a review. Semin Fetal Neonatal Med. (2012) 17:163–9. 10.1016/j.siny.2012.02.003 22364677

[11] Domellöf M Jonsson B. The Swedish approach to management of extreme prematurity at the borderline of viability: a historical and ethic perspective. Pediatrics. (2018) 142:S533–8. 10.1542/peds.2018-0478C 30171138

[12] Allotey J Zamora J Cheong-See F Kalidindi M Arroyo-Manzano D Asztalos E. Cognitive, motor, behavioural and academic performances of children born preterm: a meta-analysis and systematic review involving 64 061 children. BJOG. (2018) 125:16–25. 10.1111/1471-0528.14832 29024294

[13] Anderson P Doyle LW. Cognitive and educational deficits in children born extremely preterm. Semin Perinatol. (2008) 32:51–8. 10.1053/j.semperi.2007.12.009 18249240

[14] Anderson P Doyle LW. Neurobehavioral outcomes of school-age children born extremely low birth weight or very preterm in the 1990s. JAMA. (2003) 289:3264–72. 10.1001/jama.289.24.3264 12824207

[15] Mathewson KJ Chow CHT Dobson KG Pope EI Schmidt LA Van Lieshout RJ. Mental health of extremely low birth weight survivors: a systematic review and meta-analysis. Psychol Bull. (2017) 143:347–83. 10.1037/bul0000091 28191983

[16] Hoff B Hansen BM Munck H Mortensen EL. Behavioral and social development of children born extremely premature: a 5-year follow-up. Scand J Psychol. (2004) 45:285–92. 10.1111/j.1467-9450.2004.00407.x 15281917

[17] Weisglas-Kuperus N Koot HM Baerts W Fetter WP Sauer PJ. Behaviour problems of very low-birthweight children. Dev Med Child Neurol. (1993) 35:406–16. 10.1111/j.1469-8749.1993.tb11662.x 7684346

[18] McIntyre LL Blacher J Baker BL. The transition to school: adaptation in young children with and without intellectual disability. J Intellect Disabil Res. (2006) 50:349–61. 10.1111/j.1365-2788.2006.00783.x 16629928

[19] Fevang SKE Hysing M Markestad T Sommerfelt K. Mental health in children born extremely preterm without severe neurodevelopmental disabilities. Pediatrics. (2016) 137:e20153002. 10.1542/peds.2015-3002 26944946

[20] Gardner F Johnson A Yudkin P Bowler U Hockley C Mutch L. Behavioral and emotional adjustment of teenagers in mainstream school who were born before 29 weeks' gestation. Pediatrics. (2004) 114:676–82. 10.1542/peds.2003-0763-L 15342838

[21] Johnson S Hollis C Kochhar P Hennessy E Wolke D Marlow N. Psychiatric disorders in extremely preterm children: longitudinal finding at age 11 years in the EPICure study. J Am Acad Child Adolesc Psychiatry. (2010) 49:453–63.e1. 10.1097/00004583-201005000-00006 20431465

[22] Belfort MB Rifas-Shiman SL Sullivan T Collins CT McPhee AJ Ryan P. Infant growth before and after term: effects on neurodevelopment in preterm infants. Pediatrics. (2011) 128:e899. 10.1542/peds.2011-0282 21949135 PMC3182845

[23] Casey PH Whiteside-Mansell L Barrett K Bradley RH Gargus R. Impact of prenatal and/or postnatal growth problems in low birth weight preterm infants on school-age outcomes: an 8-year longitudinal evaluation. Pediatrics. (2006) 118:1078. 10.1542/peds.2006-0361 16951001

[24] Cooke RWI Foulder-Hughes L. Growth impairment in the very preterm and cognitive and motor performance at 7 years. Arch Dis Child. (2003) 88:482–7. 10.1136/adc.88.6.482 12765911 PMC1763118

[25] Linthavong O O'Shea TM Allred E Perrin E Bauserman M Joseph RM. Neurocognitive and health correlates of overweight and obesity among ten-year-old children born extremely preterm. J Pediatr. (2018) 200:84–90. 10.1016/j.jpeds.2018.05.011 29960765 PMC6109604

[26] Euser AM de Wit CC Finken MJ Rijken M Wit JM. Growth of preterm born children. Horm Res. (2008) 70:319–28. 10.1159/000161862 18953169

[27] Hack M Weissman B Borawski-Clark E. Catch-up growth during childhood among very low-birth-weight children. Arch Pediatr Adolesc Med. (1996) 150:1122–9. 10.1001/archpedi.1996.02170360012002 8904851

[28] McLoyd VC. Socioeconomic disadvantage and child development. Am Psychol. (1998) 53:185–204. 10.1037/0003-066X.53.2.185 9491747

[29] Noble KG Houston SM Brito NH Bartsch H Kan E Kuperman JM. Family income, parental education and brain structure in children and adolescents. Nat Neurosci. (2015) 18:773–8. 10.1038/nn.3983 25821911 PMC4414816

[30] Potijk MR de Winter AF Bos AF Kerstjens JM Reijneveld SA. Behavioural and emotional problems in moderately preterm children with low socioeconomic status: a population-based study. Eur Child Adolesc Psychiatry. (2015) 24:787–95. 10.1007/s00787-014-0623-y 25293643

[31] van Houdt CA van Wassenaer-Leemhuis AG Oosterlaan J van Kaam AH Aarnoudse-Moens CSH. Developmental outcomes of very preterm children with high parental education level. Early Hum Dev. (2019) 133:11–17. 10.1016/j.earlhumdev.2019.04.010 31035105

[32] Achenbach TM Rescorla LA. Manual for the ASEBA School-Age Forms & Profiles. Burlington, VT: University of Vermont, Research Center for Children, Youth and Families (2001).

[33] Statistics Sweden SUN, Svensk utbildningsnomenklatur [Swedish Standard Classification of Education, MIS 2000:1, SCB Sweden (2000). Available online at: http://www.scb.se.

[34] Bland JM Altman DG. Multiple significance tests: the bonferroni method. BMJ. (1995) 310:170. 10.1136/bmj.310.6973.170 7833759 PMC2548561

[35] Cole TJ Bellizzi MC Flegal KM Dietz WH. Establishing a standard definition for child overweight and obesity worldwide: international survey. BMJ. (2000) 320:1240–3. 10.1136/bmj.320.7244.1240 10797032 PMC27365

[36] Gu H Wang L Liu L Luo X Wang J Hou F. A gradient relationship between low birth weight and IQ: a meta-analysis. Sci Rep. (2017) 7:18035. 10.1038/s41598-017-18234-9 29269836 PMC5740123

[37] Luu TM Ment LR Schneider KC Katz KH Allan WC Vohr BR. Lasting effects of preterm birth and neonatal brain hemorrhage at 12 years of age. Pediatrics. (2009) 123:1037–44. 10.1542/peds.2008-1162 19255037 PMC2651566

[38] Farooqi A Adamsson M Serenius F Hägglöf B. Executive functioning and learning skills of adolescent children born at fewer than 26 weeks of gestation. PLoS ONE. (2016) 11:e0151819. 10.1371/journal.pone.0151819 26999522 PMC4801389

[39] Farooqi A Hägglöf B Serenius F. Behaviours related to executive functions and learning skills at 11 years of age after extremely preterm birth: a Swedish national prospective follow-up study. Acta Paediatr. (2013) 102:625–34. 10.1111/apa.12219 23458380

[40] Jaekel J Wolke D Bartmann P. Poor attention rather than hyperactivity/impulsivity predicts academic achievement in very preterm and full-term adolescents. Psychol Med. (2013) 43:183–96. 10.1017/S0033291712001031 22608065

[41] Van Baar AL Vermaas J Knots E de Kleine MJK Soons P. Functioning at school age of moderately preterm children born at 32 to 36 weeks' gestational age. Pediatrics. (2009) 124:251–7. 10.1542/peds.2008-2315 19564307

[42] Berglund SK Chmielewska A Starnberg J Westrup B Hägglöf B Norman M. Effects of iron supplementation of low-birth-weight infants on cognition and behavior at 7 years: a randomized controlled trial. Pediatr Res. (2018) 83:111–8. 10.1038/pr.2017.235 28953856

[43] Serenius F Ewald U Farooqi A Fellman V Hafström M Hellgren K. Neurodevelopmental outcomes among extremely preterm infants 6.5 years after active perinatal care in Sweden. JAMA Pediatr. (2016) 170:954–63. 10.1001/jamapediatrics.2016.1210 27479919

[44] Lenfeldt N Johansson A-M Domellöf E Riklund K Rönnqvist L. Alterations in white matter microstructure are associated with goal-directed upper-limb movement segmentation in children born extremely preterm. Hum Brain Mapp. (2017) 38:5051–68. 10.1002/hbm.23714 28685893 PMC6867172

[45] Peterson BS Vohr B Staib LH Cannistraci CJ Dolberg A Schneider KC. Regional brain volume abnormalities and long-term cognitive outcome in preterm infants. JAMA. (2000) 284:1939–47. 10.1001/jama.284.15.1939 11035890

